# Co‐designing a telepractice journey map with disability customers and clinicians: Partnering with users to understand challenges from their perspective

**DOI:** 10.1111/hex.13919

**Published:** 2023-11-21

**Authors:** Cloe Benz, William Scott‐Jeffs, Jerah Revitt, Chloe Brabon, Chloe Fermanis, Melanie Hawkes, Catherine Keane, Robert Dyke, Samantha Cooper, Matthew Locantro, Mai Welsh, Richard Norman, Delia Hendrie, Suzanne Robinson

**Affiliations:** ^1^ School of Population Health Curtin University Bentley Western Australia Australia; ^2^ Rocky Bay Mosman Park Western Australia Australia; ^3^ Deakin Health Economics Institute for Health Transformation, Deakin University Burwood Victoria Australia

**Keywords:** co‐design, disability, journey map, patient and public involvement, peer researcher, telepractice

## Abstract

**Introduction:**

Telepractice has the potential to align with the directive to reduce inequalities by United Nations Sustainable Development Goal 10. Telepractice additionally addresses a national digital health strategic plan for accessible digitally enabled models of care. To plan improvements, it is essential to understand the experience of telepractice for people with disability, which may be achieved through an approach such as journey mapping. The current article provides both a disability‐specific case study and a methodological guide for the inclusion of customers and clinicians in the meaningful redevelopment of services. The Perth, Australia‐based case study aimed to gain insights into the experience of telepractice for people with disability. The methodological aim describes using co‐design to produce a journey map in collaboration with customers and clinicians, for potential replication in a wide range of health and social care contexts.

**Method:**

Interview transcripts gathered from a cohort of customer participants (*n* = 17) were used to inform the journey map. A group of customers (*n* = 5) and clinicians plus one manager (*n* = 5) distributed the findings onto a customer experience journey map during a co‐design workshop. The journey map describes the emotional experience and actions taken, along five phases of a timeline through telepractice service interactions: (1) before, (2) selecting telepractice, (3) telepractice preparation, (4) during telepractice sessions and (5) after.

**Results:**

A journey map visualisation of customer experiences was produced that identified strengths of telepractice service delivery (flexibility) while noting challenges (with technology) as opportunities for improvement. The consensus of participants was the desire to have access to telepractice currently and in the future, in addition to in‐person delivery.

**Conclusion:**

These findings are valuable in the context of advocating for the incorporation of customers and clinicians through co‐design workshops in the content analysis and creation of a journey map that is representative of the lived experience of accessing telepractice services.

**Patient or Public Contribution:**

The paper forms part of a larger co‐design process that included customer participants throughout the design and planning of the project, inclusion of a peer researcher and the co‐designers in the workshops, journey map and this article production.

## 1 INTRODUCTION

Telepractice and other services, which have been rapidly implemented, upscaled or trialled, require review and potential redesign to become high‐quality sustainable long‐term services. As these service models progress from temporary responses to coronavirus disease 2019 (COVID‐19) to sustained services, they have the potential to align with the directive to reduce inequalities by United Nations Sustainable Development Goal 10.^1^ The progress of telepractice additionally works towards addressing a national digital health strategic plan for accessible digitally enabled models of care.^2^ Telepractice is defined here as services provided by a clinician to a recipient via synchronous or asynchronous digital communication means.^3^ This study focuses on the experience of people with a disability accessing clinical services delivered through synchronous telepractice via a video call.

Journey mapping is a suitable approach to use for understanding the experience of people with disability utilising telepractice as it provides a ‘visual presentation of the complete route a patient follows during all stages of a care trajectory and the patient's emotional experience through this journey’^4^ ^,p.1071^. Emotional experiences are important, as they may have a significant impact on repeat use or recommendations for telepractice to user networks. Journey mapping provides the opportunity to identify touchpoints or potential pain points within their experience to highlight areas for improved service delivery.^5^

The current study was based at a disability support service provider in Perth, Australia, providing services predominantly funded by the National Disability Insurance Scheme (NDIS). The NDIS is an Australian federal government‐funded programme, providing personalised funding plans to people with significant or permanent disability to access support via a goal‐based model.^6^ A myriad of services that are available to access under the NDIS include allied health (e.g., physiotherapy, occupational therapy, speech pathology, dietetics), behaviour support and nursing care.^7^ NDIS participants are able to utilise their plan within a fixed period (1–3 years) to receive services within categories, with each service (e.g., occupational therapy) allocated a maximum charging rate.^8^

Significant challenges of telepractice use at a population level for NDIS participants were published by Lawford et al.,^9^ which indicated that problems and barriers exist at scale. These findings, however, provided insufficient details to understand service‐level specifics. An in‐depth understanding is crucial in the pursuit of targeted telepractice innovation. Individuals who access disability services are a vast and heterogeneous group. Also, while small‐scale in‐depth inquiries could provide a level of understanding of barriers and challenges, it would be difficult to assume generalisation to the international disability community.^10^ A solution may be providing a method for replication where small‐scale in‐depth inquiries could be conducted within many local community contexts. Therefore, the purpose of publishing this article is twofold: to provide in‐depth findings to those in a similar context and to provide a methodological guide for a broader audience who may, with adaptations, replicate the process in a wide range of health and social care service improvement contexts.

The recipient and provider experiences of navigating telepractice allied health therapy services fundamentally differ. Therefore, an evaluation must appraise both customer and provider perspectives of potential improvements.^11^ Co‐design is a methodology for providing insights and guiding service design or redesign in a nonhierarchical, power‐sharing, creative thinking and doing process.^12^ It values forming relationships and building the capacity of community participants.^13^

Increasingly, research studies in health and disability have selected the co‐design methodology in advocating for more community‐based participatory research (CBPR) in its varying formats.^14^, ^15^, ^16^, ^17^ A scoping review of journey mapping use in health care published by Joseph et al.^18^ advocated for shared decision‐making, including designing in unison and constructing the journey map in partnership with service users. The Davies et al.^5^ scoping review reported that 76.5% (*n* = 62) of included studies were published since 2015, indicating increasing awareness of journey mapping strategy use to assist in centring user experience in service improvement.

The Davies et al.^5^ scoping review established eight distinct reasons justifying the use of journey mapping, the most frequent being to inform service redesign or improvement. The majority of the 26 studies additionally advocated for the inclusion of insights from people with lived experience and service providers in the journey mapping process.^5^

Correspondingly, this study aimed to gain insights into the current experience of telepractice‐delivered clinical services for people with a disability and their families with a secondary aim to explore whether empowering people with lived experience and clinicians as co‐designers to analyse data and to compile a journey map would provide useful insights to guide a telepractice service redesign.

## 2 METHODS

### 2.1 Study design

**Figure 1:**
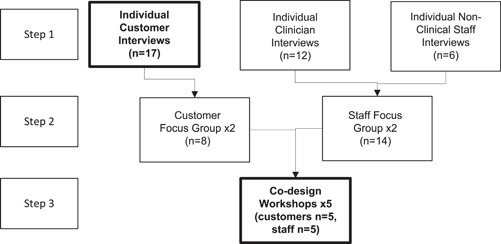
Flowchart of the project structure (items related to the current study are in bold).

The study was based on a three‐step co‐design process (Figure 1) including interviews with customers and staff, followed by focus groups to review interview findings, and ending with co‐design workshops with a smaller group of customers and staff (referred to as co‐designers). This study describes the development of a journey map based on the customer interview data and focus group discussions produced by co‐designers in the second of a five‐workshop series (step 3), with support from the workshop facilitators (C. Benz/W. S. J.). A total of 10 co‐designers who completed the series of five fortnightly workshops were recruited from the customers, clinicians and nonclinical staff interviewed in step one, who also completed a corresponding focus group. The interview participants (step 1) were offered the opportunity to express their interest to participate in the co‐design workshop series (step 3). Of those who expressed interest, selection of the co‐designers was predicated on the intent to have, at a minimum, equal number of customers to staff or preferably more customers than staff.

The project is approached in a CBPR^19^ format that was ethically governed by a set of principles prioritising lived‐experience participants as advocated for by Page^20^ and outlined by McKercher.^13^ People with a disability, carers and staff were engaged equitably throughout the project from conception to conclusion. A project steering committee contributed to study design, defining objectives, identifying participant cohorts and the recruitment and inclusion processes. A CBPR approach and **c**o‐design methodology were selected to collaborate equitably with people with a disability^21^, and a peer researcher (W. S. J.) with lived experience of disability was integral to all steps of the study. It was intentional to share power over decision‐making and project control with a member of the disability community; his involvement included, but was not limited to, the ethics application, creation of interview prompts and the journey mapping process.

Person‐first language conventions are utilised in deference to the preference of experts with lived experience who contributed to this research, to respect and affirm their identity. However, we respect the right of participants to choose and the potential for the alternate preference of identity‐first language by members of the disability community.^22^

### 2.2 Ethical approval and data reporting

Full ethical approval was received from the Curtin Human Research Ethics Committee (HRE2021‐0731). All participants provided written informed consent before the interview, and workshop attendees additionally provided specific signed consent before attendance. The reporting of the project has been completed under the guide of the Standards for Reporting Qualitative Results.^23^

### 2.3 Sampling and setting

A not‐for‐profit disability support service provider was the study setting, with inclusion criteria being customers currently accessing clinical services from the organisation and/or family members and carers (inclusive of support and education staff). Customers were purposely sampled for maximum variation in reference to disability type, age and area where services were accessed (across metropolitan, suburban and rural). Recruitment strategies included an emailed invitation, and a phone call from the peer researcher to confirm receipt of the invitation. Additionally, clinical staff who participated in interviews were asked to circulate study information and researcher contact details. Participants volunteered by contacting the research team either in person, by phone or by email and completing the consent process. They consented to either video, audio or written interview data collection and the option of a support person being present. Identification numbers were allocated for analysis and quotations, with all names and potentially identifying information replaced or omitted to safeguard anonymity.

### 2.4 Participant characteristics

All eligible customers (as of July 2022) from the study site database received an email invitation providing the opportunity to participate (*n* = 2480). A total of 19 participants consented to an interview and 17 were completed within the timeframe of recruitment from a maximum of 20. Twenty was set as a cutoff to ensure suitable numbers for subsequent focus groups (maximum 10) and workshop sessions.^24^ One participant withdrew before the interview due to health concerns and a second withdrew postinterview, with their data removed as requested, due to participant consent being withdrawn by a guardian. Participant characteristics are outlined in Table 1 and included five males and 12 females, ranging in age from 14 to 64 years, with all disclosing that English was spoken at home. All answers were optional, and participants could choose whether to disclose.

**Table 1:** Participant characteristics.

Gender	Birth year	Ethnicity	Where do you live?	Education level	Services accessed (type)	Disclosed diagnoses or impairment	Technology devices owned	Internet connection self‐rating	Participated in Telepractice
Male	1959	Caucasian	Suburban	University	Dietician, physio, OT	Other neurological, other physical, visual	Tablet	Excellent	Yes, regularly
Male	1976	Other	City	University	Physio, OT	Intellectual disability, neurological	Laptop, smart phone	Good	Yes, not many
Male	1994	Caucasian	Suburban	Tertiary certificate	Physio, OT, speech	Physical	Laptop, computer, tablet, phone	Poor	Yes, regularly
Female	2009	Asian	Suburban	High school	Physio, OT, speech	Physical	Laptop, smart phone	Good	Yes, regularly
Female	1999	Caucasian	Suburban	Tertiary certificate	Physio, OT	Neurological	Laptop, smart phone	Excellent	Yes, regularly
Female	1992	Caucasian	Suburban	Less than high school	OT, behaviour support	Autism	Smart phone	Good	No
Female	1979	Caucasian	Suburban	University	Physio, OT	Neuromuscular	Laptop, smart phone	Good	Yes, regularly
Female	1988	Caucasian	Suburban	Tertiary certificate	Physio, OT, speech	Autism, developmental delay	Laptop, computer, tablet, smart phone	Good	Yes, regularly
Male	‐	Caucasian	Suburban	Tertiary certificate	Physio, OT	Brain/spinal injury	Laptop, computer, tablet, smart phone	Good	Yes, not many
Female	‐	Middle Eastern	City	University	Physio, OT, speech, behaviour support	Autism	Tablet	Good	Yes, not many
Female	1979	Caucasian	City	University	Physio, OT, nurse	Physical	Computer, smart phone	Good	Yes, not many
Male	‐	Caucasian	Suburban	Tertiary certificate	Physio, OT	Brain/spinal injury	Laptop, tablet, smart phone	Good	Yes, not many
Female	1977	Aboriginal	Suburban	Tertiary certificate	Physio, OT	Intellectual disability, neurological, hearing loss	Tablet, smart phone	Good	No
Female	1981	Caucasian	Suburban	Tertiary certificate	Physio, OT, speech	Cerebral palsy, brain/spinal injury	Laptop, smart phone	Good	Yes, not many
Female	‐	Caucasian	Suburban	Tertiary certificate	Physio, OT, speech, psychology	Autism, anxiety, ADHD	Computer, smart phone	Good	No
Female	1986	Caucasian	Rural	High school	Physio, OT	Cerebral palsy	Laptop, tablet, smart phone	Poor	Yes, regularly
Female	‐	Caucasian	Suburban	University	N/A^a^	N/A^a^	Tablet, laptop, smart phone	Good	N/A^a^

Abbreviations: ADHD, attention deficit hyperactive disorder; OT, occupational therapy; physio, physiotherapy; speech, speech pathology.

^a^ Teacher of a student who completed telepractice sessions at school in the classroom.

### 2.5 Data collection

In step one of the co‐design process, semistructured interviews were conducted by the Peer Researcher (W. S. J.) with all customer participants supported by the embedded researcher (C. Benz), who is a registered physiotherapist, but had not worked in disability previously. Interviewees could choose an in‐person or online (via Microsoft Teams) interview at the most convenient time slot for them from the available days of the peer researcher.

Interviews were completed between July and October 2022, with participant responses related to any previous experiences of telepractice not limited to a specific timeframe. However, most participants had only experienced telepractice following the onset of the COVID‐19 pandemic, placing their experiences from March 2020 onwards.

The journey map is derived from the customer interview data set (*n* = 17), with both customer (*n* = 5) and staff (*n* = 5) co‐designers participating in the analysis of data. Focus group content was not explicitly used in the completion of the journey map; however, as each co‐designer completed an interview and focus group session, they may have implicitly used knowledge gained through those sessions.

### 2.6 Data analysis

The interview transcripts were reviewed by the first author (C. Benz) with excerpts placed on a draft timeline of the customer telepractice journey. The timeline was broken down into five phases, and all relevant interview excerpts were allocated as *doing* or *feeling* quotes. During the second co‐design workshop (2/5), the session facilitator (C. Benz) outlined the purpose, described the process of interpreting interview excerpts into components of the journey map and demonstrated completing the first phase (before) on the timeline. The co‐designers were divided into four groups and allocated a journey map phase and corresponding interview excerpts. Each group interpreted the interview comments into two categories: first, *doing*, which included strengths of telepractice and the challenges, and second, the *feeling* portrayed by the excerpts, which included a word summary and ranking out of five from thumbs down (1) to thumbs up (5). Workshop two concluded with each group feeding back and discussing their findings with the wider group before returning their responses to the facilitators. The responses were subsequently compiled into the journey map template and circulated to the group as a draft for further feedback, and all co‐designers confirmed acceptance before finalisation.

### 2.7 Journey map visualisation structure

An experience journey map of multiple customers accessing a variety of services delivered by telepractice through one organisational provider^18^ was the selected format. Alternate options such as customer journey mapping of a singular customer, a service blueprint or spatial map were deemed either too specific or broad for the intended purpose.^18^ The structure of the journey map visualisation was a flowchart with a chronological timeline that was subjected to qualitative data analysis. The care path for customers included a descriptive and visual representation of emotional experiences complementing more process‐driven actions,^4^ as emotional experiences were highlighted as a key element of the patient experience identified by the Joseph et al.^18^ scoping review.

## 3 RESULTS

### 3.1 Journey map

**Figure 2:**
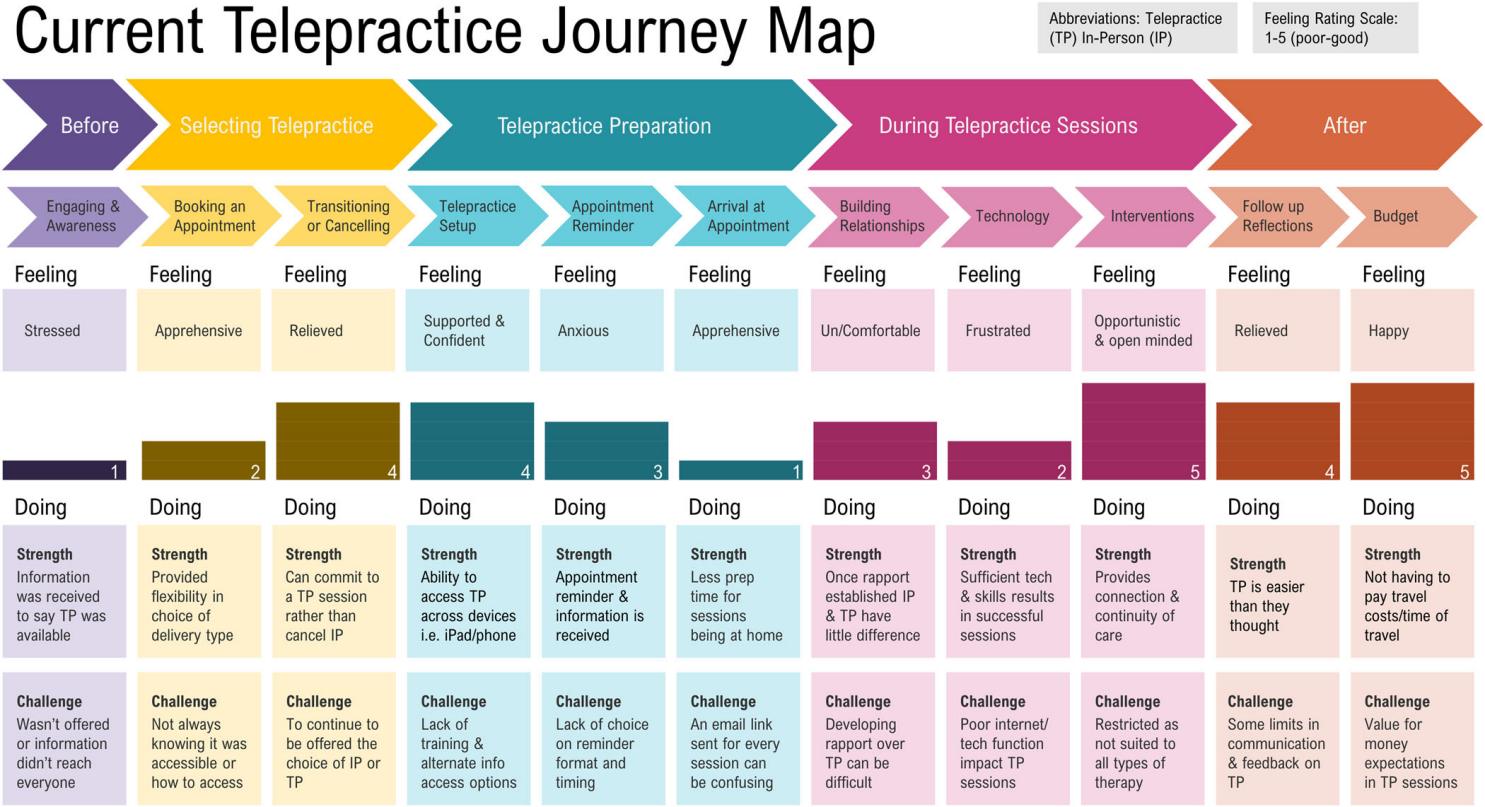
Journey map visualisation.

The co‐designed telepractice journey map (Figure 2) described five phases of telepractice therapy services, which include (1) before, (2) selecting telepractice, (3) telepractice preparation, (4) during telepractice sessions and (5) after. Each phase was broken down into components of that experience, where feelings experienced by a customer, the strengths of doing telepractice and its challenges are described. Each phase is described in more detail with supporting quotations below.

#### 3.1.1 Phase 1: Before

The emotional state of participants before encountering telepractice was described predominantly in negative terms such as stressed, confused and sceptical of the NDIS; these factors presumedly placed participants in a less open mindset.

I have many services with many different people, and it gets confusing (SU002)

A lack of positive emotions associated with the scheme that provides funding for services may have had the potential to impact uptake or receptiveness to information regarding telepractice.

Strengths and challenges identified in phase one (before) were linked to awareness of telepractice as a delivery option, with positives identified for those who were informed of its introduction and challenges seen in reaching all customers. Five participants identified the COVID‐19 pandemic as the catalyst for their awareness of telepractice, with two other participants discussing emails received offering telepractice (in response to the COVID‐19 pandemic) as their source of information. However, one participant disclosed a desire to access services via telepractice but a lack of opportunity to do so:

Never really had telepractice offered, but if it was offered, we would use it. (SU10)

Co‐designers identified the challenge of providing appropriate communication and ensuring that all potential customers were informed as a priority opportunity for future improvement.

#### 3.1.2 Phase 2: Selecting telepractice

The feeling used to summarise the selection of telepractice delivery for therapy and booking a session was apprehension predominantly linked to limitations in choice caused by the COVID‐19 pandemic. There were multiple respondents with comments such as:

It was a last resort option at the time … because of the lockdown. (SU13)

Trialling any new aspect of therapy, be it delivery mode or type of intervention, has the potential to cause apprehension if not supported sufficiently. Co‐designers and participant interviews highlighted challenges in providing sufficient information and accessibility options to support customers to feel comfortable trialling telepractice. However, telepractice provided the flexibility to transition between delivery types that decreased lost therapy time to those with mobility challenges, full‐time working parents and families who wished to holiday.

Lots of families travel these days and you don't want to be restricted because you've got a child that requires therapy, I know we've been hesitant to travel at times. If it (telepractice) became a more viable option … I think it's fantastic. (SU18)

In reference to transitioning to telepractice or cancelling appointments, relief was felt by participants as the option of telepractice was preferrable over cancelling in‐person sessions. Challenges remain regarding the best context to offer choices between in‐person and telepractice, and how to continue to offer options without overburdening the customer.

Even without the pandemic if telepractice was offered I would have chosen it in lots of sessions. (SU12)

The timing and frequency of providing the choice of delivery type were flagged by co‐designers as challenges for providing ideal access to telepractice.

#### 3.1.3 Phase 3: Telepractice preparation

The preparation for a telepractice session was broken down into three subphases, including set‐up, appointment reminders and arriving (virtually) at an appointment. Across the subphases, the emotional response from participants peaked in positivity during telepractice set‐up. Feelings were characterised as supported and confident, and progressively declining to negative due to anxiety caused by lack of reminders and apprehension before commencing a session. These feelings of anxiety and apprehension were particularly exacerbated if the provider was running late for a session:

and the person in control of the appointment is running like 15‐30 min late it starts to build up and you wonder what's going on (SU03)

During the telepractice set‐up subphase, participants valued access across a variety of devices, but flagged the need for more comprehensive training and information across options. Many participants acknowledged a level of prior understanding of videoconferencing platforms through employment and other services. However, barriers to access exist such as younger people not having email addresses, phone stands and accessories for participants with limited dexterity and portable devices to capture nimble infants moving around.

My son already has a laptop … I helped him (with setup), because he doesn't have an email to open a link. (SU13)

Appointment reminders were valued by participants who received them; however, this process was inconsistent. Within the group, the variety of preferences for reminder format and timing was viewed by the co‐designers as a key challenge:

Maybe a text message might be a bit easier, because if I get a text message it comes through straight away but with an email, I don't always get a notification. (SU08)

Telepractice appointment arrival occurs independently, unlike when attending at a therapy session in‐person and interactions occur with reception staff, or if the clinician is travelling to a customer home. The responsibility to find appropriate links and log in was a challenge; co‐designers highlighted that many people with disability access multiple services within and across organisations and links can be a real barrier and source of anxiety.

I've probably got 100 links sitting in my inbox and I don't even know which ones to click sometimes. (SU05)

The real strength of telepractice and point of difference compared to in‐person sessions was decreased travel, petrol costs and time commitments of participants. This was additionally equated to decreased impacts on both school and work attendance.

Definitely takes a lot of stress off me having to drive as well. (SU11)

A parent similarly highlighted that their adolescent son being able to access therapy via telepractice after school independently was convenient, with both parents working full time.

#### 3.1.4 Phase 4: During telepractice sessions

The three different subphases of *During Telepractice Sessions* reflect important aspects for achieving success, including building relationships, technology and the interventions to be completed.

Co‐designers linked feelings of comfort to the process of building relationships and identified that participants felt uncomfortable in the initial rapport‐building period if completed via telepractice. However, once the relationship had formed and comfort levels increased, telepractice was not significantly different to in‐person interactions. This was reflected in the identified strengths and challenges of building relationships via telepractice, with one participant describing strategies to increase tolerance for interacting on video and another suggesting that these skills were beneficial and transferrable.

The therapist and I came up with a plan to try and get him onto (video) chats with his cousins, his Aunt V and his Nana … and he thoroughly enjoys it now. (SU11)

Where technology initially created the feeling of frustration linked to multiple different factors (e.g., Wi‐Fi outages), it became an opportunity to learn new skills for telepractice sessions.

They did have a lot of trouble with sound in the beginning, they couldn't get the sound to work for quite a few sessions, which made it really difficult for them. (SU19)

Participant comments regarding technology did indicate that they felt that their confidence in using the technology grew, and they presently felt quite happy using telepractice:

Took us a little while to get our sea‐legs, but once we worked out the routine it has just been cruisy. (SU06)

Multiple carer participants acknowledged that the young person with a disability they were supporting had superior technological skills, and they were often learning rather than teaching. When technology functions appropriately and skills of users match the requirements of the session, co‐designers felt that sessions were successful. However, if either the technology or the skill set of users was insufficient, it caused significant challenges that impacted therapy sessions.

In terms of providing and receiving a diverse array of therapy interventions, co‐designers identified that participants felt open‐minded and opportunistic to trial telepractice. The COVID‐19 pandemic limiting viable alternatives may have been the source; however, this open‐mindedness enabled a baseline understanding of telepractice and its capabilities.

Telepractice enabling continuity of care during periods of stress or when in‐person sessions are not viable was a relief to many participants, with co‐designers acknowledging that COVID‐19 was unlikely to be the only cause for disruption of in‐person therapy delivery.

Having the opportunity to still be able to connect with therapists is amazing and not having to change, that's a big thing just continuing ongoing care. (SU18)

Unfortunately, despite telepractice offering a good alternative, challenges remain for the practicability of more physical interventions such as occupational therapy and physiotherapy being delivered via telepractice. However, even though exclusive use of telepractice was not desired, participants did describe enabling strategies such as alternating delivery modes, support person for hands‐on activities and utilising props to model interventions to limit the challenges faced. One participant, after acknowledging the challenges of physical interventions, described thinking that in‐person sessions with no physical component felt like a waste of travel time:

(when) there's not a physical component to it and with a lot of speech there's not a physical component, a lot of the time it feels even more of a waste going in when I could have just done it by telehealth. (SU05)

#### 3.1.5 Phase 5: After

Upon reflection posttelepractice, participants described a feeling of relief and appreciation. They felt decreased anxiety and stress knowing that telepractice was an option and wished that it would continue to be in the future:

I would love to see more of it because I think it's an integral part (of life) these days. (SU002)

Participants described a sense of surprise that telepractice was easier than anticipated, with many commenting that it is ‘not as scary as it seems’ [SU11 and SU12), they were ‘surprised how well it worked’ (SU19, SU13 and SU03) and that the advice they would give to others was to ‘go for it’ (SU02, SU03, SU06, SU13 and SU14).

Upon reflection, one challenge of increasing independence through telepractice for young people was decreased communication avenues between clinicians and the family unit:

I haven't really spoken to the OT since he started telepractice, she communicates mostly through the school but I'd prefer more direct communication and feedback. (SU19)

The final component of the telepractice journey map was on budgetary considerations, with the co‐designers deeming that participants felt happy with the impact of telepractice. Decreased travel from telepractice‐delivered services was a predominant theme, with transport costs considered to be a burden:

It's cheaper, I don't have to pay someone to travel here to my house and that's one thing I really don't like … I'd have to get someone to drive me to an appointment so that costs more as well. (SU14)

However, there were some concerns regarding value for money for specific therapy disciplines and the transferability of interventions to telepractice. In the context of funding for therapy that was provided by the NDIS scheme, this was not viewed as significant; however, one participant acknowledged that it would be more of an issue in a fee‐for‐service set‐up:

If it was out of pocket, I would probably never do OT or physio telehealth. But I'd happily spend my full money on speech telehealth. (SU05)

The overall flow of the journey map highlights some significant areas of strength for telepractice in the context of disability‐specific allied health therapy delivery and similarly identifies areas of potential improvement and current challenges.

## 4 DISCUSSION

Customer participants identified strengths of telepractice service delivery in the findings, while noting challenges as opportunities for improvement. The consensus of participants was the desire to have access to telepractice currently and in the future, in addition to in‐person delivery. The emotional experience of using telepractice fluctuated throughout the customer journey, with emotions generally more positive towards the end, compared to the outset.

The flexibility of telepractice was an identified strength, which was viewed as optimal when adjunct to in‐person services through a hybrid model, rather than as an exclusive replacement. Many participants, through this study (Phase 4: During Telepractice Sessions) and others, have identified building rapport with new providers via telepractice as difficult and preferring in‐person meetings before potentially transitioning to a hybrid model.^25^, ^26^

The need for support with technology was a challenge of telepractice described in Phase 3 (preparing for the appointment) and similarly identified in a study by Lawford et al.,^9^ in which only half of the respondents found technology easy to use and one‐fifth found it difficult. This difficulty corresponded with the most negatively rated emotional response (apprehensive, 1/5) for customers once they had commenced interacting with telepractice, which is indicative of increased support needs. The variety of challenges identified by the co‐designers in each phase reiterated the need for improvement, with Phase 5: After emphasising a desire for ongoing use.

The technological learning curve of accessing services was noted as a challenge initially for customers and potential cause for negative emotion during the preparation and completion of telepractice sessions (Phases 3 and 4). This was not unexpected considering that adults with disabilities have been shown to access internet services and use internet‐accessible devices at lower rates than adults without disabilities.^27^ Technological advancements have the potential to resolve challenges and limitations in therapy access, while simultaneously exacerbating knowledge and skill gaps that prevent people with a disability accessing services.^9^

Opportunities for accessing knowledge and supported upskilling were focuses for both interview participants and co‐designers throughout the entire journey map. A lack of technology‐specific format and adult educational pathways may be a barrier, with targeted and accessible learning pathways as a potential solution. A study by Portillo‐Aceituno et al.^26^ highlighted that a lack of digital knowledge caused parents of children with a disability to feel afraid of telepractice, with the authors advocating for specific training for parents and therapists. Multiple studies have referenced the need for training for people with a disability and therapists providing services,^28^ including two identified through using journey mapping processes.^29^, ^30^ The need for further learning opportunities similarly reiterates the questions of equitable access and the digital divide as an important issue in the context of innovation in disability and health care. However, importantly, while the digital divide is not a novel concept, this study provided co‐designers a platform to promote the need for supported education of their community rather than relying on external research assumptions.

Successful study design implementation resulted in the production of a co‐designed journey map of the current state of telepractice delivery. Meaningful, nonhierarchical partnerships between customers, clinical and management staff from an industry organisation and researchers enabled a shared decision‐making approach to the construction of the journey map, reflective of the process recommended by Joseph et al.^18^ The scoping review additionally noted that journey mapping, while showing promise, was an underutilised resource in the redevelopment of caring services.^18^ This study proposes that in combination with co‐design principles, journey mapping visualisations can advance knowledge and translate it into practice in meaningful ways for customers and providers.

### 4.1 Limitations

As Lid^10^ outlined following the release of the Convention on the Rights of Persons with Disabilities by the United Nations,^31^ in catering for specific individuals with unique needs and context, there is the potential to increase barriers for others. Also, as engaging all perspectives is difficult, including the voices of some participants may unintentionally create changes that decrease accessibility to telepractice for others. The authors acknowledge that customer co‐designers may inherently prioritise data that aligned to their lived experience and beliefs, and this highlights the point additionally made by Lid^10^ of the need for clinician inclusion. The rationale for including clinicians is their broader understanding of a wider range of disability experience at a macro‐ and meso‐level.^10^ These points, combined with the described importance of lived‐experience inclusion, emphasised the need for group collaboration across clinicians and customers, as was facilitated within the co‐designer group of this study.

The transferability^32^ of findings specific to the study context may limit direct comparison to alternate locations, contexts and times; however, opportunities exist to extrapolate the findings to guide future telepractice policy, implementation or investigations. The study publication intended to meet the dual purpose of outlining findings, but more importantly, describe a method used for others to potentially create site‐specific learnings. It guides readers in avenues of inquiry rather than dictating that findings are directly transferrable to alternate locations and contexts.

### 4.2 Future directions

This study provides the opportunity to create and implement a site‐specific telepractice improvement plan that addresses challenges currently experienced by participants. Future plans exist within the participating provider to transfer findings into meaningful change, and improvement for the customer experience, including ongoing incorporation of the project co‐designers. Study publication aimed to guide other industry and academic organisations through potential methods of co‐design and journey mapping to integrate service users. The growing demand for inclusion by community groups in research and service design innovations that directly impact them creates the need for practical examples of co‐designing in settings such as with the disability community.

On a wider scale, ongoing development of policy and guidelines inclusive of the disability community and other disadvantaged groups is required. They need to ensure that the technology and virtual care expansion does not continue to perpetuate long‐term access and equity divides. Government initiatives for internet provision through provider partnerships that incentivise network access in rural or regional areas or subsidies for people with disability as suggested by Norman et al.^27^ are a potential example. An additional alternative is inclusion of internet access in disability support payments or insurance schemes (NDIS) as the exclusion of internet services disproportionately impacts access for those who already experience disadvantage.

## 5 CONCLUSION

The current study advocates for the incorporation of co‐designers in the analysis of interview data and creation of a journey map that is representative of the lived experience of utilising telepractice at a disability support service provider. It advocates in support of both the Davies et al.^5^ and Joseph et al.^18^ scoping reviews: that customer journey mapping is a valuable tool to integrate the customer experience into service improvement and redevelopment.

The journey highlights both strengths and challenges of telepractice, with access to knowledge and supported upskilling of technology viewed as a priority throughout the entire journey. The fluctuating nature of the emotional experience of using telepractice additionally indicates areas where support is required to maintain emotional well‐being. These findings are valuable in support of the participants' desire to access a hybrid of telepractice and in‐person sessions into the future and in support of customer experience integration in the planning, design and redevelopment of the services that they access.

## AUTHOR CONTRIBUTIONS

Cloe Benz and Mai Welsh liaised with the steering committee to conceive the study structure. William Scott‐Jeffs and Cloe Benz developed the initial draft of the interview schedule, which was revised by Mai Welsh, Suzanne Robinson, Delia Hendrie and Richard Norman. All of the above contributed to the ethical application and protocol development. Cloe Benz and William Scott‐Jeffs completed the recruitment and data collection as well as the data allocation to the journey map timeline. In their role as co‐designers Jerah Revitt, Chloe Brabon, Chloe Fermanis, Samantha Cooper, Catherine Keane, Matthew Locantro, Melanie Hawkes and Robert Dyke completed the data analysis and completion of the journey map visualisation. Cloe Benz wrote the first draft of the manuscript. All authors reviewed and edited the manuscript and approved the final version of the manuscript.

## CONFLICT OF INTEREST STATEMENT

The authors declare no conflict of interest.

## ETHICS STATEMENT

This study was approved by the Curtin University Human Research Ethics Committee (ID# HRE2021‐0731). All participants of the study provided written informed consent before participation, with the co‐designers providing additional informed consent for the co‐design workshops.

## Data Availability

The data that support the findings of this study are available on request from the corresponding author. The data are not publicly available due to privacy or ethical restrictions.
